# Delivery of Distance Counselling to Survivors of Sexual Violence: A Scoping Review of Promising and Best Practices

**DOI:** 10.1177/00469580221097427

**Published:** 2022-05-05

**Authors:** Janette Leroux, Natalie Johnston, Ashley-Anne Brown, Alanna Mihic, Denise DuBois, AnnaLise Trudell

**Affiliations:** 1 Sexual Assault Centre Kingston, Kingston, Ontario, Canada; 2 School of Rehabilitation Therapy, 4257 Queen’s University, Kingston, Ontario, Canada; 3 Department of Public Health Sciences, 4257 Queen’s University, Kingston, Ontario, Canada; 4 Dalla Lana School of Public Health, 7938 University of Toronto, Toronto, Ontario, Canada; 5 Anova, Gender-Based Violence Shelter and Sexual Assault Centre London, Ontario, Canada

**Keywords:** distance counselling, telehealth, videoconferencing, sexual violence, gender-based violence, scoping review

## Abstract

Distance counselling holds immense potential for improving access to trauma supports for survivors of sexual violence (SV), and particularly for under-served groups who disproportionately experience violence and myriad barriers to accessing in-person supports. And yet, the evidence-base for the practice and delivery of distance counselling remains under-developed. In the context of COVID-19, where telehealth applications have undergone a rapid uptake, we undertook a scoping review of existing evidence of therapeutic and organizational practices related to the real-time (synchronous) delivery of distance counselling to survivors of SV. We based our scoping review methods on Arksey and O’Malley framework and in accordance with the guidance on scoping reviews from the Joanna Briggs Institute (JBI) and PRISMA reporting guidelines for scoping reviews. A comprehensive search of MEDLINE, Embase, PsycINFO, CINAHL, Web of Science, and Sociological Abstracts was undertaken in October 2020, and again in March 2022. Searching, reviewing, appraisal, and data extraction was undertaken by two reviewers. In total, 1094 records were identified that resulted in 20 studies included. Descriptions, findings, and recommendations were gleaned and synthesized into potential practices using inductive thematic analysis. While many studies have an appreciative orientation to distance counselling, these benefits tend to be framed as non-universal, and conditional on survivor safety, flexibility, anonymity, survivor choice, strong and inclusive technology, and a supported workforce.

Despite the limited evidence-base, we present several clusters of findings that, taken together, can be used to support current COVID-19 distance counselling initiatives with survivors, as well as guide the future development of best practices.

**What do we already know about this topic?**

Telehealth applications such as distance counselling hold immense potential for alleviating mental health burden but previous to the pandemic, remained largely under-explored and under-utilized for survivors of sexual violence, who experience unique therapeutic needs and barriers to accessing supports.

**How does your research contribute to the field?**

Our findings outline the merits and cautions of distance counselling for survivors of sexual violence, as well as several important design considerations for the development of a distance counselling program.

**What are your research’s implications towards theory, practice, or policy?**

In the context of a rapid and urgent transition to provision of supports by distance due to the COVID-19 pandemic, this review increases the accessibility and practicability of practice- and evidence-informed guidance related to the delivery of distance counselling to survivors of sexual violence. Our findings also point to future areas of research, including wider representation of survivor populations, broader conceptualization of access, investigation into harmful effects and therapeutic alliance, as well as new therapeutic possibilities made possible by distance technologies.

## Introduction

Sexual violence (SV) refers to any sexual act when consent is not obtained or not freely given.^1^ SV encompasses a range of survivor-perpetrator relationships that involve different forms of coercion and contexts of vulnerability.^1^ Sexual violence is pervasive. It is estimated that one out of three women and one out of eight men will experience SV in their lifetime.^1^ Vulnerability to sexual violence is shaped by intersecting structural and political contexts of racism, poverty, and sexism.^2,3^ In Canada, women, young people, First Nations/Metis/Inuit, single, LGBQ, those with mental health challenges, those who have experienced childhood abuse and homelessness, and individuals who have more evening activities outside the home report the highest rates of sexual assault.^4^

The model of provision of support to survivors of SV tends to be community-based crisis and trauma counselling, and advocacy. In Canada, there exists a rich network of women’s shelters and sexual assault/rape crisis centres (SAC/RCCs) across the country.^5,6^ Despite being chronically under-funded, and heavily relying on non- and under-paid work of staff and volunteers, these community-based agencies provide vital resources for survivors.^7^

Distance communication technology holds promise in the gender-based violence (GBV) sector, in terms of improving cost-effectiveness of service provision and facilitating wider access to counselling supports. There is increasing demand for and expectation of communication technologies, among survivors who may face considerable financial, geographical, physical, or social barriers to accessing supports in person.^8^

The therapeutic experience of survivors of sexual trauma necessitates consideration in the context of counseling by distance, including safety, privacy, and security, which serve as precursors to the formation of a strong therapeutic alliance needed for healing.^9,10^ For many survivors, accessing counselling supports can feel like outing oneself.^11^ Survivors often report feelings of shame, fear, guilt, and self-blame due to the stigma surrounding SV.^12^ Services and supports offered to survivors should be responsive to their needs, trauma-informed, and not revictimizing,^13^ independent of delivery modality.

Counsellors and advocates have had several justifiable concerns regarding the risks and potential harms of distance counselling for survivors. Some of these concerns include the limits to privacy and security by distance, concerns about the ability to form a strong therapeutic alliance by distance (missing non -verbal cues), being disconnected from the supportive environment of the actual agency centre, and an overall prevailing belief that distance counselling services are inferior to face-to-face counselling modalities.^8,14^ These concerns among others, along with the precarity of working conditions in the GBV sector (ie heavy caseloads, staff turnover, undercompensated, vicarious trauma and burnout), and concomitant lack of evidence-based practices or practice-based evidence, are likely contributors to the reticence of agencies and counsellors alike to implement distance counselling programs.

Indeed, leading up to the COVID-19 pandemic, there had been limited literature on the effectiveness or special considerations of distance counselling for survivors of SV.^14^ With the disastrous onset of COVID-19 through all global continents in the winter/spring of 2020, physical distancing pandemic countermeasures forced widespread agency closures and rapid transition to providing distance supports. Rather than being a modality of exception, distance care quickly shifted to being an opt out rather than opt in.

This took place against a backdrop of the historical precarity of funding within the gender-based violence sector, where survivor-serving agencies have always struggled to meet the demand within their communities on insufficient funding.^15^ The implications for such being outlined in other literature, but of relevance to the inquiry at hand: staff burnout and turnover, and consistent under-investment in operational equipment including the IT infrastructure and training for staff that would be requisite to quickly implement a robust distance counselling program. Without this IT infrastructure in place, alongside a precariously employed workforce, delayed acknowledgment of the essentiality of services provided by SAC/RCCs, and a lag in emergency funding, volunteers and staff across the GBV sector struggled to move towards providing distance counselling safely, efficiently, and exclusively. The conditions for providing distance counselling were shifted into counsellors’ homes, the exacting challenges and consequences of that being described elsewhere.^16^

SV is notably different from other forms of violent trauma, through socio-cultural mechanisms of stigma, victim-blaming and silencing, and disempowerment,^17^ which limits the transferability of research on distance therapy among other patient populations who have suffered trauma.^18-20^ We differentiate synchronous distance counselling from information and communication technologies (ICT) that are asynchronous, for example, web-based self-help treatments,^21^ mobile apps for health (mHealth) addressing one-time emergency or avoidance solutions, screening, or decision-making aids,^22^ prevention intervention,^23^ or the delivery of health services.^24^ Recognizing the sparseness of research on distance counselling for survivors of SV specifically, alongside the widespread, urgent need for evidence- and practice-based guidance on how to provide trauma counselling safely and effectively by distance, we undertook a scoping review of academic and clinical literature to gather information regarding the real-time (synchronous) delivery of distance counselling for survivors of sexual trauma. Our specific research question was,

“What are the therapeutic and organizational practices related to the synchronous delivery of distance counselling to survivors of sexual violence?”.

In describing the existing literature, we had the following three specific objectives for our study: I) Provide a broad overview of the research to date, including the various distance parameters, models of delivery, survivor populations, and research and clinical findings; II) Identify gaps in the literature, in order to suggest future areas of study for this particular inquiry; III) Glean practices, lessons learned, recommendations, next steps, and any inferred insights, from interpreted findings, so as to synthesize and extrapolate into a practical series of best and potential practices for agencies and staff currently engaging in distance counselling in the present context of COVID-19.

## Method

The methodological framework for this review was based on the Joanna Briggs Institute (JBI) Methodology for JBI Scoping Reviews.^25^ This method builds on the original scoping review framework developed by Arksey and O’Malley.^26^ Scoping reviews employ systematic literature searching, screening, and analysis, and are aimed at mapping key concepts, types of evidence, and gaps in research relating to a research or conceptual area.^25^ Scoping reviews are different from systemic reviews in that they provide a comprehensive overview of the area addressing broader review questions.^25^ For a nascent or scattered body of literature, a scoping review can serve to assess the quantity and breadth of current research, and direct attention to existing gaps as opposed to appraising individual pieces of literature, and generalizability and strength of evidence.^26^ Scoping reviews are becoming an increasingly common approach to informing non-clinical programs in community-based settings, where ontological and epistemological underpinnings of research and practice do not necessarily fit with the traditional hierarchy of clinical evidence.^27^

### Search Strategy

We consulted with a research librarian to develop and pilot our search strategy, including the development of subject headings and search terms. A three-step search strategy was used in this review: 1) Our formal search included published studies in the following 6 different electronic databases: MEDLINE (Ovid), Embase, PsycInfo, CINAHL, Web of Science, and Sociological Abstracts; 2) Subsequent searching included screening references of eligible studies; and 3) Hand-searching selected journals – 6 journals on telehealth (Telemedicine and e-Health, Journal of Telemedicine and Telecare, Journal of Medical Internet Research, International Journal of Telemedicine and Applications, Telemedicine Journal, Internet Interventions) and 3 journals on SV (Trauma, Abuse and Violence, Violence Against Women, Journal of Gender Based Violence). All search returns were uploaded to COVIDENCE, the collection of which was automatically de-duplicated. Two independent reviewers screened all titles and analysed the relevance of the articles to be included in the review based on the information provided in the title and abstract. The full-text version was obtained for all studies meeting the inclusion criteria for our review. The two reviewers then independently examined the full-text version to determine whether they met the inclusion criteria. Any disagreements between reviewers were resolved through discussion. Preliminary searching, returns from hand-searched journals, and decisions around operational concepts and definitions are detailed in Supplemental Appendix A. Specific search record including search terms and subject headings as used in PsycInfo is detailed in Supplemental Appendix B. Our original search was conducted in October 2020 and updated in March 2022.

### Inclusion and Exclusion Criteria

Following JBI guidelines, this scoping review considered any article type including research articles, clinical case studies, commentary or program reports, and any methodology including quantitative studies, including experimental or observational study designs, qualitative studies, and literature reviews. This review was undertaken without any date restrictions. Due to the limitations of our study team, only articles published in English were included in this review. We included any peer-reviewed literature published on survivors of any form of SV. We excluded articles that described preventive distance counselling programs for sub-populations deemed vulnerable to SV. We also excluded articles that focused on abusive men or perpetrator populations, and cybersex addicted adults. We excluded articles that focused on non-adult populations, unless it related to mother and child. We excluded articles that covered asynchronous distance therapy, including online learning psycho-education. We excluded articles that focused on hotlines or crisis lines, one-off consults, or physical nurse examinations. We excluded articles that focused on decision-making tools and apps, including mHealth and eHealth, as they have been described elsewhere.^21-24^ Furthermore, we excluded articles where the online or distance aspect of the article related to research data collection rather than being the therapeutic modality itself.

### Data Extraction

A table was developed by researchers to extract the most relevant details for our analysis. This descriptive data included: article type, aim of article, survivor population, distance setting, and methodological design and data collection. Within an extension of this table, we also created a coding system to extract statements, findings, or interpretations of distance counselling, from the vantage of survivor, counsellor, and organization. In addition, based on these vantages, we extracted descriptions of distance counselling conditions, insights into uses of technology, and any exclusion criteria for participating in distance counselling. Two reviewers extracted all data and assigned codes independently. Any disagreements were resolved through discussion. Each reviewer independently charted each study using the data extraction form created in COVIDENCE. We then met to review our results and come to a consensus with any inconsistencies.

### Data Synthesis

The collection of articles was described as a whole by: country, article type, article focus, survivor population, and distance setting. The coded qualitative data gleaned from each article was then distilled into short statements and labelled by article. From there, all statements were re-read in entirety, and were grouped and re-grouped by emergent themes. Researchers read and reviewed the original statements and their location and fit within thematic groups. Researchers then came up with potential practices for each theme, by original category (survivor, counsellor, organization), and discussed as a group. Each of these potential practices were then back-checked, and verified against sub-theme and theme, as well as distilled statement, categorized findings, and original context within each article. Lastly, each article was re-read, alongside the practices indicated, to discover any contradictions or contrary findings against these practices within the overall collection of literature.

## Results

### Study Selection

**Figure 1.**
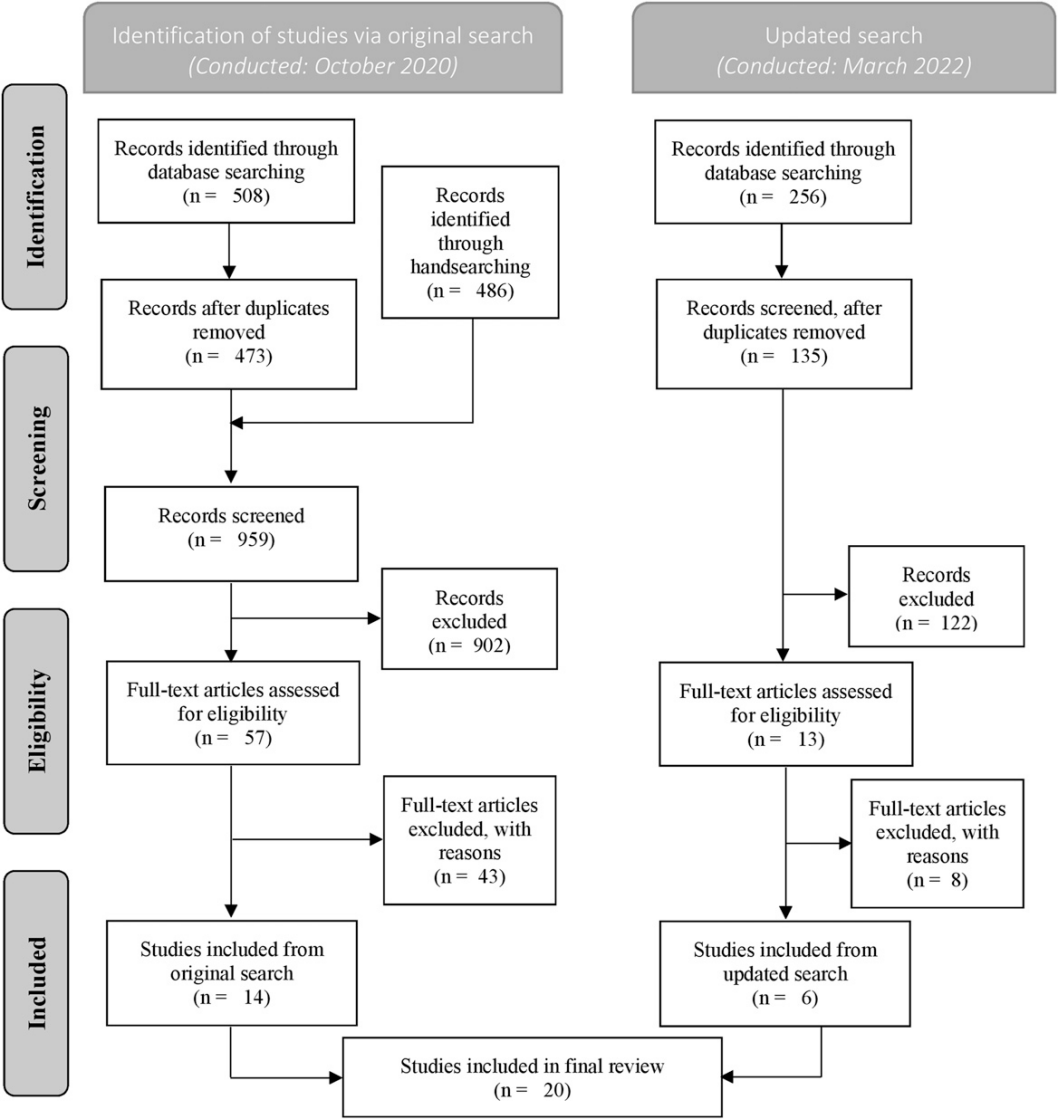
PRISMA flow diagram for process of identification and inclusion of studies. *Adapted from:* Moher D, Liberati A, Tetzlaff J, Altman DG, The PRISMA Group (2009). Preferred Reporting Items for Systematic Reviews and Meta-Analyses: The PRISMA Statement. PLoS Med 6 (7): e1000097. doi:10.1371/journal.pmed1000097.

For the original search conducted in September 2020, a total of 508 records were retrieved through searching 6 different electronic databases. Two independent reviewers screened all records, and assessed 57 full-text articles for eligibility, of which 12 articles met the inclusion criteria (Kappa score of .59). Reference mining of eligible studies yielded 21 records. Hand-searching specialized gender-based violence journals and telemedicine journals yielded 465 records. From these records, 2 met inclusion criteria, bringing the total articles to 14 (Figure 1). The same process was replicated with our updated search conducted in March 2022. A total of 256 articles were identified and yielded 6 additional studies to be included (Kappa score of .47), bringing the total of our overall collection of articles to 20.

### Descriptive Summary of Studies

A descriptive summary of the 20 included articles, including article type, study aim, survivor population, distance setting, and methods used are reported (Table 1). All literature arose from the United States, except for two recent studies from Australia. Most studies were focused on survivors of military sexual trauma or domestic/intimate partner violence. Four studies focused on rural access. While five studies described experimental comparisons between distance modalities,^28-32^ only one study was a true randomized controlled trial.^33^ Most articles were descriptive, including a book chapter, four commentaries, two case studies, and five clinical program evaluation studies. Seven of the articles focused on evaluative dimensions of distance counselling, including utility, feasibility, acceptability, effectiveness, attrition, and preferences. Several studies employed pre-post treatment comparisons of symptom scales to determine effectiveness of distance counselling. Additional measures included patient satisfaction, trainee and staff satisfaction, attendance, session participation attrition (drop-out), and modality preferences. We found few explicit references to therapeutic alliance as a concept,^34^ and no studies directly measured therapeutic alliance as a construct.

**Table 1.** Summary of articles included in the current study.

Article Type	Aim	Survivor Population *(sample size)*	Distance Setting	Methods	
				Design	Data Collection
Azevedo et al, 2016 - *Piloting Specialized Mental Health Care for Rural Women Veterans Using STAIR Delivered* via *Telehealth: Implications for Reducing Health Disparities*					
Descriptive (Evaluation)	• Determine whether implementation of STAIR program reduce PTSD and increase social engagement	Women veterans with military-related trauma who live in rural areas (n=17)	• Clinic-to-clinic setting or clinic-to-home videoconferencing	• 10-week skills building pilot program delivered via telemental health	• Therapist reported via weekly meetings
					• Patients complete weekly skills exercises
	• Describe experiences testing feasibility, acceptability of telemental mental health program to rural women veterans		• Group (90 mins) or Individual (45 mins)	• Client choice of group or individual format, at home or at satellite outpatient clinic	• Questionnaires: Patient responses to open-ended questions
					• Patient satisfaction survey
Baffsky et al, 2022 - *“The real pandemic’s been there forever”: qualitative perspectives of domestic and family violence workforce in Australia during COVID-19*					
Qualitative Research article	• Understand DFV service responses to pandemic	Domestic and family violence practitioners (n=51)	• Digital mode of service delivery	• Observational analysis of frontline DFV service providers in different contexts	• Semi-structured interviews with DFV workers
	• Inform future effective practices		• Remote working		
Banducci, 2021 - *Prolonged Exposure Therapy in the Time of COVID-19: Modifying PTSD Treatment for a Military Sexual Trauma Survivor Who Contracted COVID-19 Mid-Treatment*					
Case study	• Describe modifications made due to pandemic and telehealth transition	Military sexual trauma (n=1)	• Telehealth: 3 initial sessions to complete psycho-diagnostic assessment, 16 sessions of PE (90 mins, 1-2 times per week)	• Description of telehealth treatment considerations at patient-, provider-, and systems level	---
	• Convey considerations for PE as distance treatment modality				
Cortis et al, 2021 - *Adapting Service Delivery during COVID-19: Experiences of Domestic Violence Practitioners*					
Mixed methods Research article	• Examine service responses (experiences, adaptations, innovations) to pandemic	Domestic and family violence service providers (n=100)	• Remote, technology-mediated modes	• Observational analysis of practitioners in DFV not-for-profit community-based organisations	• Survey with multiple free-text questions
	• Consider long-term implications for service delivery		• Working at home and in isolation		
Emuzue, 2021 - *Digital or Digitally Delivered Responses to Domestic and Intimate Partner Violence During COVID-19*					
Commentary	Rapidly describe current DV mitigation approaches using digital solutions; emerging best practices to support survivors, their children, abusers during stay-at-home advisories	Women and girls who experience violence by an abusive partner in intimate and casual relationships (n=N/A)	Digital or digitally-delivered health interventions	---	---
Gilmore et al, 2016 - *“Do you expect me to receive PTSD care in a setting where most of the other patients remind me of the perpetrator?”: Home-based telemedicine to address barriers to care unique to military sexual trauma and veterans affairs hospitals*					
Descriptive (Evaluation)	Comparison of feasibility and efficacy for in-person vs HBT for female veterans with MST	Female veterans with military sexual trauma related PTSD (n=100)	• VAMC or affiliated satellite clinics	• Description of federally funded ongoing RCT comparing prolonged exposure (PE) delivered in-person to PE delivered via HBT	• Process outcomes: Session attendance, service satisfaction, QOL indices
					• Intent-to-treat strategy
					• Variables examined as confounders and associated w/drop-out
			• 12-week active intervention phase (weekly sessions)		• Hypotheses tested at 3 post-treatment timepoints (GLMM)
					• Effect sizes for PTSD symptoms (CAPS, PCL)
					• Thematic interviews (qualitative analysis for treatment drop out and non-responders)
Gray et al, 2015 - *Provision of evidence-based therapies to rural survivors of domestic violence and sexual assault* via *telehealth: Treatment outcomes and clinical training benefits*					
Evaluation	Preliminary examination of benefits, impacts of telehealth treatment from three stakeholder groups: Psychology doctoral student therapists, crisis center staff, clients	Rural survivors of sexual assault and domestic violence (n=21)	• WTTTC and satellite sites (domestic violence/rape crisis centers)	• Evaluate client response to telehealth treatment (PTSD and depression symptom reduction) plus client satisfaction	• Non-randomized (client modality preference), pre/post intervention: Symptom measures (PTSD, CES-D, WTTCCSS- Satisfaction)
				• Examine perspectives, perceived benefits by trainees, crisis center staff	• Training benefits – satisfaction scale
					• Crisis centre staff – satisfaction ratings
Hassija and Gray, 2011 - *The effectiveness and feasibility of videoconferencing technology to provide evidence-based treatment to rural domestic violence and sexual assault populations*					
Evaluation	Evaluate feasibility, effectiveness of videoconferencing-administered, trauma-focused treatment among rural domestic violence, sexual assault survivors presenting to community assault, domestic violence crisis centers	Female victims of assaultive violence (n=15)	• WTTTC and satellite sites (domestic violence/rape crisis centers)	• Pre-post treatment comparison: Participants were referred (non-randomized), ≥4 sessions by videoconferencing from distal site (center)	• Measures: PTSD symptom severity (PCL), depression symptom severity (CED-D), WTTCCS client satisfaction questionnaire
				• Weekly sessions, 60-90 mins, assessments every 4 sessions	
Jarnecke and Flanagan, 2020 - *Staying safe during COVID-19: How a pandemic can escalate risk for intimate partner violence and what can be done to provide individuals with resources and support*					
Commentary	Examine strategies to increase IPV safety during salient periods of increased stress exposure now and in future	Situational couple violence (less frequent and severe IPV) and intimate terrorism (n=N/A)	• Telehealth	---	---
Kaukinen, 2020 - *When Stay-at-Home Orders Leave Victims Unsafe at Home: Exploring the Risk and Consequences of Intimate Partner Violence during the COVID-19 Pandemic*					
Commentary	Explore potential short- and long-term implications of COVID-19 on the risk of IPV, highlighting some of the most recent preliminary data	Women experiencing violence by an intimate partner (n=N/A)	• Telehealth	Discussion on impacts of crime and violence on women during COVID-19 pandemic; differential impacts on vulnerable populations, including minority women and those with long histories of victimization and mental health issues	---
Morland et al, 2019 - *What do veterans want? Understanding veterans' preferences for PTSD treatment delivery*					
Quantitative Research article	Assess veterans preferences for treatment delivery modalities and how demographic variables and trauma type impact these preferences	Male and female veterans with PTSD (n=180). Of the study sample 29% had experienced MST	• Veterans affairs Hospital, Community based outpatient clinics, Veterans’ homes	Observational analysis to examine differences among modality preferences and participant characteristics	• Descriptive statistics, chi-square tests, and regression analysis, using treatment delivery modality questionnaire (HBT, OBT, IHIP) and demographics variables (questionnaire)
Ragavan et al, 2020 - *Supporting Adolescents and Young Adults Exposed to or Experiencing Violence During the COVID-19 Pandemic*					
Commentary	To recommend a course of actionable, trauma-sensitive practices to support adolescents and young adults' (AYA) unique needs and challenges	AYA exposed to or experiencing violence (parental or caregiver IPV, adolescent relationship abuse, or youth violence) during COVID-19 (n=N/A)	• Virtual relationship-building	Recommendations for actionable, trauma-sensitive practices to address AYA unique needs and challenges	• Vignettes with potential response and resource provision
					• Practical theoretical application: CUES (Confidentiality, universal Education, Empowerment, and Support) - evidence-based approach that prioritizes offering information, resources to all AYAs during clinical encounters
Sitz et al, 2021 - *Cognitive Processing Therapy With an Older Woman Veteran During COVID-19: A Case Study*					
Case Study	• Provide evidence for effectiveness of CPT in telephone-based setting	Military sexual trauma (n=1)	• Telephone-based telehealth	Description of telehealth treatment, process, and clinical considerations	---
	• Provide guidance for best implementing CPT-like treatments with clients unable to do video				
Steinmatz and Gray, 2017 - *Treating emotional consequences of sexual assault and domestic violence* via *telehealth*					
Book Chapter	Describe videoconferencing-based technology as a means of connecting rural trauma survivors with psychological care, to alleviate mental healthcare disparities that result from rurality	Female Sexual assault and domestic violence survivors (women aged 19-52) (n=N/A)	• WTTTC and satellite sites (domestic violence/rape crisis centers)	Narrative	• Chapter sections: Training required (treatment providers and IT support); Pros and Cons of clinical setting; Evidence base for interventions described
			• ≥ 6 weekly sessions, 60-90 mins		
Stevens et al, 2015 - *A Trial of Telephone Support Services to Prevent Further Intimate Partner Violence*					
Quantitative Research article	Investigate utility of interventions to address IPV	English-speaking women ages 18 years and above, who screened positively for IPV in past year (n=26)	• 12 phone calls to total 360-720 minutes over 6 months	Randomized-controlled trial of telephone support services vs enhanced usual care	• Pre-mid-post intervention measures: IPV (Composite abuse Scale, Women’s Experience with battering Scale), relationship questions, mental health (CES-D, PCL), general and physical health questions, perceived social support (Social Provisions Scale), Effectiveness in Obtaining Community Resources (EOR Scale), adverse events questions
Thomas et al, 2005 - *Telepsychiatry Program for Rural Victims of Domestic Violence*					
Evaluation	Describe telemedicine program that provides psychiatric screening, evaluation, treatment, referral for ongoing care to clients of a rural women’s crisis center	Rural victims of domestic violence or child abuse (n=31)	• Galveston Center for Telehealth and Distance Education	• Partnership formation	• Symptom Checklist −90-R (SCL-90-R), 90-item self-report scale of psychiatric problems w/9 subscales of differing symptom clusters
			• East Texas Rural Women’s Shelter (2 shelters +6 outreach clinics)	• Descriptive analysis of diagnoses (via psychiatric evaluation)	• Physical examination, medical history, laboratory results, psychological interview
					• Satisfaction questionnaires
Valentine et al, 2020 - *Comparing PTSD treatment retention among survivors of military sexual trauma utilizing clinical video technology and in-person approaches*					
Quantitative Research article	Compare completion rates and symptom attrition of evidence-based PTSD psychotherapy delivered via CVT or in-person among veterans with PTSD receiving treatment for MST index traumas	Veterans with PTSD following MST (n=171)	• Veterans health administration or Clinical video technology	Comparison of speed of attrition of PTSD symptoms between in-person and CVT approaches	• Data collected w/routine care - MINI, DSM-IV, CAPS
					• Data analysis – demographic, service-related, clinical characteristics; associations between program completion, session attendance
Voth Schrag et al, 2022 - *“So many extra safety layers:” Virtual service provision and implementing social distancing in interpersonal violence service agencies during COVID-19*					
Qualitative Research article	Capture rapid changes to understand strategies that evolved within IPV services	Intimate partner violence, sexual assault, child abuse, human trafficking service providers (n=33)	• Virtual service provision (zoom based visits)	Explore experiences of IPV service providers implementing virtual services in pandemic	• Semi-structured interviews with anti-violence service providers with queries on demographics, experiences, client safety and service use during pandemic, agency approaches, occupational stress
Wood et al, 2020 - *On the Front Lines of the COVID-19 Pandemic: Occupational Experiences of the Intimate Partner Violence and Sexual Assault Workforce*					
Quantitative Research article	Capture evolving service delivery methods, shifting safety planning approaches, occupational stress of frontline workers	Intimate partner violence, sexual assault service providers (n=352)	• Remote service provision (telecommute or office/home) – video conferencing and video calling with clients	Explore experiences of IPV service providers implementing virtual services in pandemic	• Survey with questions on client safety and safety planning strategies, technology use before and after pandemic
Zheng and Gray, 2014 - *Telehealth-based therapy connecting rural Mandarin-speaking traumatized clients with a Mandarin-speaking therapist*					
Qualitative Research article	Address feasibility of bridging lingual divide in rural areas for non-English speakers by providing telehealth-mediated treatment to sexual assault and IPV victims who speak only Mandarin	Rural, Mandarin-speaking Chinese immigrant women who experienced sexual assault or abusive relationships (n=2)	• University of Wyoming Psychology Telehealth Clinic	Case series - 2 cases	• Account of treatment implemented • Assessment conducted throughout sessions (weekly for 7 and 17 weeks): PTSD Checklist, CESD-R (4 month follow-up), WTTCCSS- Satisfaction Scale, Subjective stress

*Note*. AYA = adolescent and young adults; CPT = cognitive processing therapy; CVT = clinical video telehealth; DFV = domestic and family violence; HBT = home-based therapy; IPV = intimate partner violence; IT = internet technology; MST = military sexual trauma; PTSD = posttraumatic stress disorder; QOL = quality of life; RCT = randomized controlled trial; PE = prolonged exposure; VAMC = Veterans Affairs Medical Center; WTTTC = Wyoming Trauma Telehealth Treatment Clinic.

In terms of the delivery conditions of distance counselling, not all articles provided explicit details on the structure of distance counselling programs. Of the articles that did, we found considerable variability in terms of timing and duration of programs. In regards to distance “model of delivery”,^35^ eleven studies described satellite scenarios, where survivors accessed services at a remote clinic that virtually connected to a more central clinical setting. Of the more recent studies published since the onset of the COVID-19 pandemic, the model of distance model of delivery described are the “clinic-to-home” or “home-to-home” model. All four commentaries, also published in that timeframe, describe an increased and intensified risk of violence and vulnerability deriving from the pandemic, and put forward various general practices and mitigation strategies for distance engagement.^36-39^ Four observational studies published most recently relay the evolution of service responses, the impact this pandemic work on frontline practitioners, and look to long-term future implications on the sector.^40-43^

### Thematic Analysis

**Figure 2.**
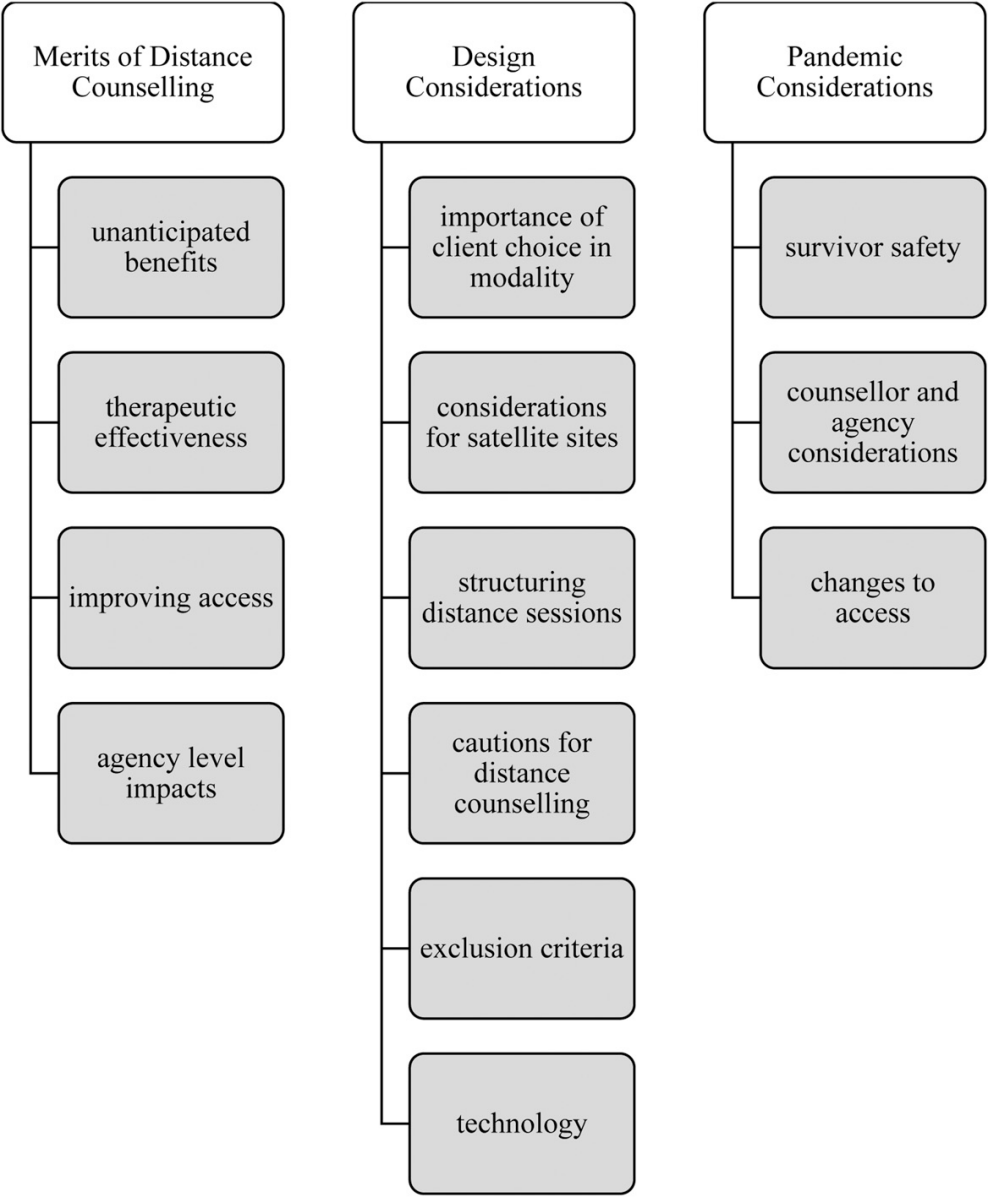
Conceptual overview of 3 major themes (white), and 13 sub-themes (grey) derived from thematic analysis of extracted statements, findings, or interpretations of distance counselling from the current collection of literature.

Our sample of extracted data yielded three thematic areas, and 13 subthemes about the conditions, parameters, impacts and effectiveness of delivering distance counselling (Supplemental Appendix C; Figure 2). The interpretations of the practical and key take-aways from these themes and sub-themes are presented as considerations and potential practices that could be adopted and implemented, to be evaluated or tested in survivor-serving agencies, along with pandemic-specific considerations and practices (Table 2).

**Table 2.** Summary of critical findings.

**Merits of Distance Counselling**
o Unanticipated therapeutic benefits of and preference for distance counselling (attributes of distance counselling that allowed for survivor control, privacy, anonymity, confidentiality and flexibility and accordingly improved the therapeutic experience)
o Therapeutic effectiveness of distance counselling, and the improvement in access to supports
o The interpretation of such findings being: do not assume distance counselling is the inferior modality (may be the only modality for some groups); Expect agency-level impacts from offering distance counselling (changed use of resources in the agency, changed referrals); Distance modality may improve access to counselling by ameliorating transportation, childcare, scheduling conflicts, language and anonymity barriers
**Design Considerations**
o Importance of client choice in modality: Survivor-centred modality choice and design will promote attendance, therapeutic alliance, sense of safety, and therapeutic effectiveness
o Emphasis on technology: Technology underpins survivor safety; secure, encrypted, HIPPA compliant software and hardware; as technology evolves must respond with resources, IT support and investment; reliability of technology is important, and several studies point to having backup contact plans in place between survivor and counsellor to minimize disruption
o Cautions for distance counselling: Some instances where distance counselling may be inappropriate (need for assessment and strong communication with each survivor to determine continued individual candidacy)
o Additionally discussed: Considerations for virtual sites, structuring distance sessions, and exclusion criteria
**Pandemic-Specific** ** Distance Conditions**
o Changes to survivor safety brought about by the pandemic conditions: Violence is transformed and intensified; new barriers to getting help
o Technology means access to supports but also introduces new and intensified threats to safety and security
o New safety considerations, given sheltering-in-place, with respect to scheduling (asking about safety and privacy), expanding IPV screening and support to everyone, and offering tech options for user security measures (stalker detection software, authentication and verification protocols)
o The agency may be the only private/safe place, and to consider keeping it open with strict protocols as a last option for the most vulnerable survivors
o Agencies cannot rely on traditional channels of mass communication and referrals and cannot assume survivor access to technology; pandemic has changed patterns of service access
o Counsellor and agency considerations included: Recognizing staff are taking on brunt of mismatch between demand and capacity for transitioning to distance, and require working flexibility, new ways for self-care, peer support, and support resources (including training, IT support, and protective equipment); nature of work has changed and intensified resulting in occupational stress, interpersonal tensions

## Merits of Distance Counselling

### Unanticipated therapeutic benefits and preferences for distance counselling

Several studies discuss unanticipated therapeutic benefits of distance counselling, for reasons including survivors feeling more comfortable and in control at home,^28,33,34^ as well as the flexibility, privacy, and level of anonymity that distance affords.^31,33,44^ Together, these statements suggest that distance delivery should be designed to promote survivor control, privacy, anonymity, confidentiality, and flexibility.

### Therapeutic Effectiveness of Distance Counselling

Studies that examined or considered the comparative effectiveness of distance counselling to face-to-face counselling reported no inferiority of therapeutic outcomes or client satisfaction,^29,44-47^ suggesting that it is distance counselling should not be assumed to be an inferior modality of delivery.

### Distance mode of delivery improves access to counselling

In this collection of studies, several statements and findings point to the ways that distance counselling may improve access to services and supports. Some studies indicate specific contextual conditions and sub-populations,^34^ while others offer general discussions of accessibility with respect to unique considerations for survivors of sexual violence.^40-42^ These insights suggest that distance counselling may be the *only* modality for some sub-groups of survivors, improving access to counselling by ameliorating transportation, childcare, scheduling conflicts, language barriers, and safety and stigma concerns of accessing in-person services. The advantages of added anonymity were especially highlighted for populations that might fear that their disclosures of violence or their physical presence at an agency could make them vulnerable to law or immigration enforcement.^42^

### Agency-Level Impacts

While many concerns and questions about delivering distance counselling tend to centre on the therapeutic interaction between practitioner and client, several studies point to the important considerations for the ways that offering a distance counselling program will change the use of resources in the agency and its very relationship within the community it serves. For example, some studies point to the ways that distance counselling increases access to services for previously under-served clients,^34,44^ impacting the referrals and use of other health and social services in the community as a whole.^34^ Other studies discuss the changed use of resources within the centre, relating to scheduling - due to flexibility and fewer cancellations^40^ - staffing, training, and technological equipment and infrastructure.^40,42,43,46^ For example, distance counselling changes the outreach and online presence of the agency necessitating attention to the “virtual front door”, and needing to place the same level of care and attention to online experience, safety and security as agencies do in-person.^42^

## Design Considerations

### Importance of client choice in model of delivery 

The importance of client choice, framed as expanded treatment options and prioritizing survivor voices and perspectives, emerged as an important theme in discussion of the design of distance counselling, in terms of improving attendance and reducing attrition.^20,28,33,36,44-46^ This was underscored by the idea that it was the survivor-centredness of distance modality as a choice and a means to address survivor barriers accessibility and inclusion that promotes attendance, therapeutic alliance, safety, therapeutic effectiveness.

### Considerations for virtual sites (including satellite sites, counselling from home) 

The details surrounding the physical and logistical setting up of virtual sites received attention in this collection of studies. Several studies involved a satellite model of delivery (ie clinic-to-satellite site), and of the studies to include descriptions of the virtual site, the warmth of satellite staff, the administrative intake and routines, and the availability of IT support were all discussed as important considerations for survivors experiences of distance counselling.^28,44^ The studies published since the pandemic, tended to describe a clinic-to-home or home-to-home delivery model, where considerations for the set up of the physical space of the counsellor as well as the client are discussed,^42,47^ as well as the mental and emotional impacts on a workforce delivering trauma counselling from home.^40-43^

### Structuring Distance Sessions

In terms of designing the parameters of distance sessions, incidental and explicit program descriptions indicate a divergence in the structuring of distance sessions. For example, suggesting a front-end loading of sessions for information gathering and rapport building.^32,44,46^ Others recommend including new approaches, such as shared-decision making tools for determining treatment plan and delivery modality,^20^ and exploring new therapeutic options made possible with distance, such as including peer support during in-vivo exercises.^32^

### Cautions for Distance Counselling

Different forms of cautionaries emerged from this collection of studies, which underscored importance of assessment and strong communication with each survivor to determine continued individual candidacy for distance modality. Some studies pointed to the role of avoidance in symptom management, as well as survivor safety and security deriving from the specific ways they have been/are being victimized by violence.^31,32,36^ Studies published since the pandemic also tended to describe other general concerns about the challenges of distance counselling, specifically in regard to assessing risks for and impacts of violence, as well as clients’ circumstances and needs, ensuring the solitary presence of survivors, the comparative quality of distance compared to in-person, as well as building rapport and catching non-verbal cues.^40-42^

### Exclusion Criteria

Pre-pandemic literature tended to detail exclusion criteria for distance counselling as follows: active psychosis or bipolar disorder, dementia, at risk of self-harm, suicidal, homicidal, or substance dependent.^30,32,34,44,45^ Since the pandemic, the discussion of inclusion in distance counselling tended to be more vague, to suggest considering which patients will persist in treatment by distance, specifically exploring with clients how trauma-focused distance counselling would fit into their day-to-day lives given pandemic stress levels.^46,47^

### Technology Discussions

Technology was given considerable attention within this collection of studies, including the specific platforms and technological capabilities, as well as IT infrastructure and digital security practices and processes. In these descriptions, technology was framed as being fundamental to survivor safety, and the importance of secure, encrypted, HIPAA compliant software and hardware, as well as strong and reliable internet connection. Specific practices and processes relating to technology were discussed, including having a backup plan for connection should the technology fail,^42,44,45^ as well as strategies to minimize the “communication trail” through which help-seeking is identifiable – including storing clients’ correspondence in de-identified way, using a protected number, avoiding the downloading of apps, and checking for software that automatically stores identifiable logs of calls between clients and practitioners.^40-43^

## Pandemic-specific Distance Conditions

### Survivor safety

Survivor safety was a central concern in all studies published since the pandemic, and was discussed in terms of: the transformed and intensified violence deriving from pandemic conditions and stressors exacerbating already existing disparities among sub-populations of survivors; new barriers to getting help due to isolation, coercive control, and changes to service provisions; and the acceleration of new forms of technology-based abuse and isolation.^36-38^ Specific practices and processes to promote survivor safety were suggested, including: expanding IPV screening and support to all clients; inquiring about safety and privacy when scheduling and initiating sessions; confirming client location at outset of each session; implementing systems of counsellor/survivor passcodes, code words to signal risks, disguised apps, personalized PIN numbers, special stalker detection software, covert authentication and verification protocols; introducing new ways to share information and seek privacy (ie chat function), being flexible with and confirming best methods for communication and sharing of materials with each client;^36,38,39,41,47^ and recognizing that the agency may be the only private or safe space available to survivor to keep doors open with strict protocols as last option for some survivors.^39^ The studies that discuss safety planning suggest that these are collaboratively developed, inherently individualized,^41,43,47^ and do not assume police or other formal first responders are safe and supportive avenues to address potentially violent situations.^43^ And still, several studies that relayed practitioner workforce experiences pointed to the rapid transition online and limited time to implement a full scope of survivor safety procedures,^41^ and further point to the challenges of preserving client privacy and confidentiality for practitioners working from home within their own pandemic experiences (ie children present, unsuitable home working environments, etc.).^41,43^

### Counselor and agency considerations

All articles published since the pandemic discuss the ways that the pandemic is impacting agencies and practitioners, with respect to the ways that the nature of the work has changed and intensified resulting in occupational stress and interpersonal tensions, as well as the ways that staff are taking on the brunt of the mis-match between demand for services and capacity for transitioning to offering distance.^36,39-43,47^ Specific concerns relate to the professional isolation and increased risk of vicarious trauma,^40^ the challenge for frontline workers to take time off putting them at risk of burnout,^40^ working extended hours to meet increasingly complex needs of growing clientele,^40^ burden to appraise and become familiar with safe use of new technologies,^36^ a lack of reference points to practical guidelines or survivor specific evidence base,^41^ against a backdrop of frequent shifts in workplace protocols amidst changing pandemic conditions.^42^ Specific mention of the new ways of working involving more “task work” related to technology,^41^ the need for new sets of resources to know and develop,^43^ more check-ins due to concerns for elevated risks posed by pandemic,^40,42^ expanded contact hours,^41^ providing additional support outside of regular sessions,^46^ and changed content of the counselling to reflect emergent or crisis needs-in-the-moment.^42,43^ Practitioners in the GBV sector tend to be an underpaid and precariously employed workforce, and much of this literature points to the importance of supporting the mental well-being of frontline staff through: intentional acts of self-care and separation from work, self-care plans, group support sessions, flexible work schedules to accommodate caregiver responsibilities, sick leave and paid time off, hazard pay, counseling, assistance with material and resource support.^36,39,42,43^

### Global changes to access

Several studies point to the ways that the shift to technology mediating the relationship between clients and supports has fundamentally changed access, and on a global scale. The implications of such are described as: not being able to rely on traditional channels of mass communication and systems of referrals, and not assuming survivors have access to technology.^37,41,42^ Other studies stress a new form of survivor advocacy for addressing the digital divide, in recognizing the overlay of inequities in digital participation and the most vulnerable survivor populations.^39-41^ In acknowledging the ways that the pandemic-induced technology shift has transformed the sector into the future, some studies suggest that virtual services will enhance the reach of IPV/SV services beyond the pandemic^43^ - especially for populations that experience the most marginalization and stigmatization from seeking services^42^ – and virtual services should be continued and potentially added as part of an “a la carte service model”.^42^

## Discussion

In this scoping review, we found a limited body of literature pertaining to the practice and delivery of distance counselling. With a wide frame to glean details and findings from this extant literature, and using inductive analysis, we were able to map out potential areas of consensus for the benefit of current survivor-serving agencies and practitioners (Table 2).

### Broad Overview of Literature to Date

Our study collection spanned a wide timeframe, including the transformative time of the early COVID-19 pandemic, and as such there tended to be methodological differences between pre/post pandemic articles, involving different distance models of delivery, and different concerns for survivors. Earlier articles overwhelmingly reported on evaluative dimensions of distance counselling among smaller candidate groups of survivors, predominantly involving satellite clinics (“clinic-to-clinic”) models of delivery and were highly attentive to a range of exclusion criteria that tend to be prevalent among survivors. This pattern of evaluative and non-experimental research reflects a cautious and slow approach characteristic of a sector that operates with extreme care to not introduce new dangers to survivors nor interfere with recovery.^48^ Later articles published since the onset of the pandemic were commentaries, two case studies, and four observational studies exploring the widespread impacts of the pandemic from the perspective of the GBV workforce and capturing the innovations and adaptations in response to the pandemic. These later studies tended to convey a “clinic-to-home” and more often a “home-to-home” model of delivery and attempted to describe a mixture of deleterious and advantageous aspects of distance counselling for practitioners and broad survivor groups.

Indeed, the challenges of trial by fire amidst a paucity of research are clearly echoed in the more recent literature.^41,49^ While the pandemic forced considerable innovation and uptake of distance models of delivery in the GBV sector, it remains unclear if and how the original concerns underpinning practitioner reticence to adopting distance counselling have been allayed. While many studies have an appreciative orientation to distance counselling, these benefits tend to be framed as non-universal. For example, the advantages of distance counselling with survivors are consistently cited as being conditional on how as a model of delivery it can promote survivor safety, flexibility, anonymity, survivor choice, strong and inclusive technology, and a supported workforce. Researchers point to the potential that continuing offering safe and effective virtual services as a complement to in-person services would have in enhancing accessibility and inclusion for the most stigmatized and marginalized populations into the future.^42,43^

### Potential Areas of Future Research

There are downsides and cautionaries for distance counselling, as were captured in thematic area of design considerations. Consistent with the nascent area of telehealth research more broadly, there has been little attention to the micro- or macro-level negative effects of distance counselling for survivors of SV. There is a growing area of interest in potential negative effects of internet interventions,^50^ with proposed investigation into the differences of negative effects between face-to-face treatment and internet interventions.^51^ In addition to investigating severe adverse events resulting from internet interventions, researchers are pointing to other types of negative effects, including deterioration, less serious adverse events, novel symptoms, dropout, nonresponse, unwanted events.^50^ Future research would benefit from using an open system lens to explore how offering distance models of delivery can carry both negative and positive impacts, on survivors, the community, and the sector.

Particularly when exploring the design of a service delivery modality intended to improve access, in order to avoid the reproducing social, cultural and structural barriers to inclusion of in-person services, it will be important for future work in this area to focus on the needs of historically under-served groups who disproportionately experience violence alongside myriad barriers to accessing in-person supports.^52^ While no studies explicitly challenge the dominant depoliticized frame of SV as an interpersonal issue, future research should acknowledge the history and ongoing reality of systems of oppression such as racism, sexism, and colonialism,^2,3^ and how these intersect to shape vulnerability and experiences of SV and exclusion from supports.^53^ In this way, distance counselling can move service-provision access to be more equitable, and resist sliding into neoliberal territory of efficiency and cost-savings.

In the current study, we gleaned and synthesized experimental findings and experiential insights in published literature, to contribute evidence-informed practice(s) of synchronous delivery of distance counselling to survivors of SV. There is much opportunity in harnessing practice-informed evidence (the flipside of evidence-informed practice), and particularly in the current scenario of scarce scholarly literature but ample “real-world” experience. The pandemic has pressed many survivor-serving agencies and practitioners to trial out different program models and practices. This presents a tremendous opportunity for future research, to capture this frontline experiential wisdom, and apply novel ways to synthesize and share such findings for broader application. In this way, mixed-method program evaluation studies could contribute important information about feasibility, acceptability and effectiveness of distance counselling among survivors, and may better fit the with ethos and understandings of violence in SAC/RCCs, as well as the limits of experimental research with survivors of SV.

There were several challenges in pulling together findings from the current collection of literature, including an overall lack of explicit details on the distance counselling conditions such as the frequency and duration of sessions, as well as the specific distance models of delivery. Future research should include rich process evaluation descriptions of the implementation and program parameters. Explicitly sharing details of the distance counselling parameters helps to safeguard against exporting status quo conventions of in-person counselling and thus limiting the therapeutic potential and possibilities of distance counselling. For example, in-person sessions are typically allotted a 1-hour appointment timeslot, however research suggests that distance sessions are naturally shorter in duration,^54^ attributable to the disinhibition effect.^55^ During the pandemic, the content of sessions during the pandemic was reported to have shifted from trauma processing to more crisis support, and the contact was shorter and more frequent.

Rich descriptions will also enable an understanding in broader terms of what may work, with whom, and where. In the current collection of literature, assumptions around normative distance models of delivery were only evident with additional investigation, which revealed eleven studies employed a “clinic-to-clinic” model for distance counselling (ie client travels to site that is set up with technology to be connected remotely to clinician). It remains unclear what role satellite models of delivery hold in improving accessibility and inclusion in service provision for survivors, but likely replicate many of the limitations of in-person care models. Clarifying the distance setting in an article is critical to the interpretation of findings, as “clinic-to-clinic” distance settings would introduce different barriers to access as well as an additional therapeutic environment to consider as compared to “clinic-to-home” or “home-to-home” arrangements. For example, emerging research on the impacts of COVID-19 on people employed in the gender-based violence sector clearly demonstrates the intensity of providing trauma counselling from home.^16^ The lack of physical boundaries between work and home, and the loss of habitual vicarious trauma prevention practices and peer support mechanisms,^56^ is an important distinction to make in existing literature, and points to an important future research direction which would explore ways to reduce the emotional burden of SV counsellors working from home.

Therapeutic alliance is generally considered to be fundamental to the effectiveness of therapy in general and is especially important in psychotherapy and counselling for survivors of SV. As described by Judith Herman,^57^ “the alliance of therapy cannot be taken for granted, is painstakingly built by the effort of both patient and therapist… to create a healing space where the therapeutic work centres on creating a sense of safety.” We found few explicit references to therapeutic alliance as a concept,^33^ and no studies directly measured therapeutic alliance as a construct. It remains unclear how therapeutic alliance may form differently, function differently, or require different conditions by distance as compared to in-person.^45^ Practitioners tend to point to several concerns around missing non-verbal cues, such as body language and eye contact, and furthermore emphasize the role that these components of the interaction play in the therapeutic use of silences and pauses which can feel confusing and ambiguous by distance.^58,59^ While there has been some investigation into therapeutic alliance by distance,^60^ and for some trauma populations,^61^ therapeutic alliance by distance for survivors of SV would entail unique elements and considerations for measurement, and overall remains entirely unexplored in existing literature.

A summary of the implications of our scoping review for future research is presented in Table 3.

**Table 3.** Summary of implications for research.

• Evaluative and non-experimental research may be most practical and suitable to this inquiry given the widespread uptake of distance counselling in the context of COVID-19, point to methods that amplify frontline experiential wisdom and survivor experiences
• Explicit descriptions of distance counselling conditions and models of delivery will be important for valid comparisons and greater opportunities for shared learning among service-providers
• In general, future research inquiries should include: Wider representation of survivor populations, broader conceptualization of access, investigation into harmful effects, therapeutic alliance, as well as new therapeutic possibilities made possible by distance technologies
• In the context of COVID-19, future research can shed light on ways to reduce emotional burden of counsellors working with a home-to-home distance counselling model

## Limitations

To our knowledge, this is the first scoping review examining distance counselling practices for survivors of SV. This is a timely and important synthesis that can guide practitioners and service providers working with survivors of SV during the COVID-19 pandemic and provides a broad overview of the literature to date including gaps and areas for research into the future.

We employed a rigorous and transparent methodological approach to undertake a systematic and comprehensive search of the literature. It is possible that we did not capture all studies pertaining to our inquiry. While we attempted to capture a diversity of terms describing distance counselling, because telehealth is a newer concept, and especially emergent within the context of the widespread uptake with COVID-19, we may have missed literature that uses other terminology. Our collection of literature spans a timeframe which includes early pandemic literature. There are fundamental differences between the methods, models, and concerns for survivors and the workforce, while we integrated findings as conjunctive takeaways, there will be other ways to compare and contrast these subsets of literature. It remains unclear how the takeaways presented will hold up, as the pandemic continues, and more knowledge is gained and literature emerges on both distance counselling and the experiences of survivors of SV in a pandemic in general. Furthermore, as is possible with any interpretive/constructivist research, other researchers may have come to different conclusions as than what we have here. While we undertook a thorough and thoughtful analysis and synthesis, and back-checked our findings with the original articles, we come to the work with our own sets of assumptions and ideas. We acknowledge the extrapolation that was necessary to extend the findings into practicable takeaways. We have documented our process and presented as much as possible in the formatting of our results.

While there exist appraisal frameworks that can account for heterogeneity in study design and methods (Mixed Methods Appraisal Tool),^62-65^ we determined to not assess the quality of evidence in the studies in this review. While appraisal of evidence could have given more or less weight to some promising practices, we felt that appraisal was an activity that did not contribute to our research question at hand. Instead, we focused on pulling narrative and descriptive evidence from all sections of each study to describe and construct practical meaning from a very dispersed and limited body of literature. Furthermore, we acknowledge the several limitations to conducting experimental research on survivors of SV (as outlined above), and accordingly chose not to create hierarchies of evidence within our study.

## Supplemental Material

Supplemental Material - Delivery of Distance Counselling to Survivors of Sexual Violence: A Scoping Review of Promising and Best Practices Click here for additional data file. Supplemental Material for Delivery of Distance Counselling to Survivors of Sexual Violence: A Scoping Review of Promising and Best Practices by Janette Leroux, Natalie Johnston, Ashley-Anne Brown, Alanna Mihic, Denise DuBois, AnnaLise Trudell in INQUIRY: The Journal of Health Care Organization, Provision, and Financing

## Appendix

### Abbreviations

APA: American Psychological Association
DV: domestic violence
GBV: gender-based violence
HIPAA: Health Insurance Portability and Accountability Act of 1996
IT: internet technology
ITC: information and communications technology
IPV: intimate partner violence
JBI: Joanna Briggs Institute
SAC/RCC: sexual assault centre/rape crisis centre
SV: sexual violence
WHO: World Health Organization.
