# Supplementary material for: Delivery of Distance Counselling to Survivors of Sexual Violence: A Scoping Review of Promising and Best Practices
Source: Inquiry. 2022 May 5;59:00469580221097427. doi: 10.1177/00469580221097427 (PMC9082742; doi:10.1177/00469580221097427)
Supplement: Supplemental Material - Delivery of Distance Counselling to Survivors of Sexual Violence: A Scoping Review of Promising and Best Practices [file sj-pdf-1-inq-10.1177_00469580221097427.pdf]

APPENDIX A.

**Preliminary Searching, Operational Concepts & Definitions**

JL and NJ consulted with a research librarian to develop and pilot our search strategy, including the development of subject headings and search terms (Appendix B). We began by collating a selection of studies and search terms. AAB and JL hand searched reference lists for snowball identification of related publications and journals. JL, AM, and DD looked at recent reviews related to telehealth, to ensure we captured relevant indexed terms and subject headings for this relatively new concept. We refined our research question using the JBI Scoping Reviews population, concept, context (PCC) research tool[25]. We determined **population** to be survivors of any form of SV, as defined above. We sought to capture the elusive **concept** of distance counselling by drawing on terms, nomenclatures and definitions used in telehealth, telepsychology, telepsychotherapy, distance therapy, online counselling, and online therapy literature. While we searched neighbouring concepts, we drew from the American Psychological Association to define distance therapy, "*any type of psychotherapy in which sessions are not conducted face-to-face… include[ing] interventions by telephone, audioconference, or videoconference and the internet*"[65]. We determined that there would be different considerations for survivors based on the parameters of our concept of distance counselling, and different broad sets of research questions around safety and therapeutic efficacy for non-person to person therapy, asynchronous interactions, crisis outreach, and psycho-education. We narrowed our concept of distance counselling according to our study objectives; particularly the third study objective. In this way, our concept of distance counselling included: person-to-person therapy, synchronous, and sustained therapeutic relationship, to reflect the predominant widespread format of uptake of distance counselling. Likewise, we considered **context** in a way that would

allow for practical insights into the feasibility and effectiveness of distance counselling, and accordingly defined context as the therapeutic and organizational practices related to the delivery of distance counselling.

To inform our review, AAB and JL conducted a preliminary search for existing related systematic and scoping reviews and protocols was carried out in October 2020, using Cochrane Database of Systematic Reviews, Campbell Collaboration Education Group, JBI Database of Systematic Reviews and Implementation Reports, EPISTEMONIKOS, and PROSPERO: International Prospective Register of Systematic Reviews. There were two reviews that were identified to have potential relevance[66,67]. However, upon closer examination, one was very broad in their concept of telehealth and not specific to survivors of SV[67], and the other was focused on a narrow and asynchronous form of distance support (Ehealth applications) for survivors of intimate partner violence[66].

**Appendix B.** Specific search strategy used in **APA PsycInfo**, from the dates 1806 to 2020 September 1. These search strings were modified as appropriate for the other databases outlined in the methods.

| # | Searches | Results |
|---|---|---|
| 1 | sexual abuse/ or incest/ or rape/ | 27721 |
| 2 | sexual harassment/ | 2611 |
| 3 | sex offenses/ | 10312 |
| 4 | sexual violence.mp. | 4596 |
| 5 | sexual* trauma*.mp. | 1694 |
| 6 | domestic violence/ | 11564 |
| 7 | domestic abuse.mp. | 761 |
| 8 | sexual assault*.mp. | 6891 |
| 9 | sexual misconduct*.mp. | 529 |
| 10 | gender based violence.mp. | 834 |
| 11 | intimate partner violence/ | 11784 |
| 12 | or/1-11 | 61924 |
| 13 | computer assisted therapy/ | 1100 |
| 14 | computer therap*.mp. | 34 |
| 15 | cyber counsel*.mp. | 15 |
| 16 | cyber psych*.mp. | 39 |
| 17 | distance counsel*.mp. | 49 |
| 18 | distance therap*.mp. | 18 |
| 19 | e counsel*.mp. | 65 |
| 20 | e therap*.mp. | 197 |
| 21 | interapy.mp. | 13 |
| 22 | e consult*.mp. | 42 |
| 23 | econsult*.mp. | 16 |
| 24 | electronic consult*.mp. | 31 |
| 25 | ehealth.mp. | 1114 |
| 26 | e health.mp. | 999 |
| 27 | evisit*.mp. | 4 |
| 28 | e visit*.mp. | 11 |

| | | |
|---|---|---|
| 29 | home video visit*.mp. | 0 |
| 30 | m health.mp. | 143 |
| 31 | mhealth.mp. | 1004 |
| 32 | offsite care.mp. | 0 |
| 33 | off site care.mp. | 1 |
| 34 | online counsel*.mp. | 308 |
| 35 | online therap*.mp. | 3071 |
| 36 | ontario telemedicine network.mp. | 7 |
| 37 | remote consultation*.mp. | 689 |
| 38 | remote visit*.mp. | 3 |
| 39 | tele care.mp. | 11 |
| 40 | telecare.mp. | 237 |
| 41 | teleconsultation/ | 64 |
| 42 | telehealth.mp. | 1777 |
| 43 | tele health.mp. | 53 |
| 44 | telemedicine/ or online therapy/ or exp teleconferencing/ or teleconsultation/ or telepsychiatry/ or telepsychology/ or telerehabilitation/ | 8889 |
| 45 | tele monitor*.mp. | 18 |
| 46 | telemonitor*.mp. | 178 |
| 47 | tele practice*.mp. | 1 |
| 48 | telepractice*.mp. | 96 |
| 49 | tele mental health.mp. | 27 |
| 50 | telepsych*.mp. | 766 |
| 51 | tele psych*.mp. | 27 |
| 52 | video consult*.mp. | 58 |
| 53 | videoconsult*.mp. | 4 |
| 54 | virtual care*.mp. | 27 |
| 55 | videoconferencing/ or video-based interventions/ | 678 |
| 56 | or/13-55 | 13177 |
| 57 | 12 and 56 | 73 |

**Appendix C.** Thematic analysis, extrapolation to considerations and potential practices.

| Theme/*Sub-Theme* | Considerations/Potential Practices |
|---|---|
| **Merits of Distance Counselling** | |
| ***Unanticipated therapeutic benefits of and preference for distance counseling*** | |
| - Closer to home and in survivor's home meant more in control, more comfortable sharing information concerning thoughts and feelings (Azevedo et al., 2016) | ⇒ **Design distance to promote survivor control, privacy, anonymity, confidentiality, flexibility** |
| - Acute needs of individuals presenting at crisis centers - many are unable to commit to extended course of therapy (Hassija and Gray, 2011) | |
| - Participant preference for telephone contact over face-to-face: flexibility in being reached, feelings of partial anonymity (Stevens et al., 2015) | |
| - Gap in recommended policy versus service delivery practices (MST has practical and financial costs for victims and families) (Gilmore et al., 2016) | |
| - More comfortable, easier to keep privacy (Zheng and Grey, 2014) | |
| - Participants were satisfied and the program was well-received (Thomas et al., 2005) | |
| - Teleconferencing increases anonymity and confidentiality (Steinmatz and Gray, 2017) | |
| - Telephone may decrease ability to assess nonverbal cues but it may also facilitate contact (Stevens et al., 2015) | |
| - Telepsychiatry may have facilitated patient comfort and alliance because it provided a level of control and distance (Thomas et al., 2005) | |
| ***Therapeutic effectiveness of distance counseling*** | |
| - Any perceived barriers or peculiarities resulting from [distance counseling] not at expense of efficacious outcomes or client satisfaction (Gray et al., 2015) | ⇒ **Do not assume distance counselling is inferior modality** |
| - [Distance counseling] capable of achieving comparable gains to in-person services (Hassija and Gray, 2011) | |
| - [Distance therapy] was effective in reducing the severity of PTSD symptoms and treatment gains were large and maintained for 4-months following therapy (Zheng and Grey, 2014) | |
| - Clients symptom improvement demonstrates modality meets needs of rural populations otherwise unlikely to receive services at all (Gray et al., 2015) | |
| - Large reductions of symptom severity following [distance] treatment, high degree of satisfaction with [distance] services (Hassija and Gray, 2011) | |
| - Evidence indicates that the gains are comparable to traditional in-person services (Steinmatz and Gray, 2017) | |
| - It is possible to continue to utilize effective, evidence-based treatments for PTSD during global pandemic (Banducci, 2021) | |
| - Evidence indicates effectiveness of CPT as services transitioned from in-person to telephone-based setting (Sitz et al., 2021) | |
| ***Distance mode of delivery improves access to counseling*** | |
| - Telepsychiatry can provide rapid crisis intervention and effective mental Health services to victims of DV in a rural setting (Thomas et al., 2005) | ⇒ **Distance counselling may** |

| | |
|---|---|
| - [Distance] may be only option for access to trauma-focused care (Valentine et al., 2020) | **be *only* modality for some groups**<br>⇒ **Distance modality may improve access to counselling by ameliorating transportation, child care, scheduling conflicts, language, anonymity barriers** |
| - Relevant comparison for rural clients is not in-person services - but rather, no psychological services at all (ability to mitigate any degree of symptoms is valuable improvement to the standard level of care) (Hassija and Gray, 2011) | |
| - Removes geographical, time, transport barriers to accessing services (Baffsky et al., 2022) | |
| - Remote service delivery enabled services to be maintained [during pandemic] and improved access for some client groups (Cortis et al., 2021) | |
| - Removes need to physically travel to agency to receive services (reduces burden of service access); without transportation, child care, scheduling conflicts, services more accessible to parents, full-time workers, those with mobility limitations, survivors fearing questions from abusive partner about their travel (Voth Schrag et al., 2022) | |
| - Facilitates language access otherwise unavailable (Voth Schrag et al., 2022) | |
| - Gives survivor ability to remain anonymous (i.e anonymous to provider or unseen accessing services) which is of benefit to survivors who fear stigma related to service seeking or fear that law or immigration enforcement could be called to an agency (Voth Schrag et al., 2022) | |
| ***Agency-level impacts*** | |
| - Teleconferencing (with satellite referral sites) increases access to mental healthcare for rural clients: minimizes geographical barriers, and for satellite agencies mitigates insufficient time to learn ESTs, lack of access to relevant information resources, and prohibitive expense of training (Steinmatz and Gray, 2017) | |
| - Program improved community mental health referrals (Thomas et al., 2005) | |
| - More flexibility with scheduling at home, not relying on availability of staffing or equipment (Azevedo et al., 2016) | |
| - The impact of successful intervention in DV and reduction in the use of emergency and in-patient psychiatric care should be taken into account in assessing the potential cost-effectiveness of this program (Thomas et al., 2005) | ⇒ **Offering distance counselling will change the use of resources in agency and its relationship within the community** |
| - Low burden, scalable in terms of reaching victim-survivors unable to be supported face-to-face; fewer cancellations attributable to ease with which clients can access treatment (Baffsky et al., 2022) | |
| - Leverage resources to problem solve around technological barriers (i.e., iPad loaner programs, etc) (Banducci, 2021) | |
| - With virtual service model, agencies need to focus on “virtual front door”, taking same care as in-person survivor comfort with additional attention to safety and digital security (Voth Schrag et al., 2022) | |
| - More flexibility in connecting with clients, can call survivors from multiple locations to open up more opportunities for advocates to connect with survivors in ways that meets their needs and eliminates barriers to help-seeking (Voth Schrag et al., 2022) | |
| - Critical need for additional training, infrastructure, support for virtual modalities (Wood et al., 2020) | |
| **Design Considerations** | |
| ***Importance of client choice in modality*** | |
| - More adherence to attendance of sessions, less trauma avoidant, more able to build skills, increased perceptions of safety (Azevedo et al., 2016) | ⇒ **Survivor-centred modality choice and design (either way) will promote attendance, therapeutic alliance, sense of safety, therapeutic effectiveness** |
| - Client preferences against [distance] may contribute to higher rates of attrition (Hassija and Gray, 2011) | |
| - Expanding treatment delivery options through a patient-centered and flexible approach may increase veterans’ willingness to seek and remain in therapy (Morland et al., 2019) | |
| - MST history did not predict treatment preferences in any analysis (both options desirable) highlighting the value of | |

| | |
|---|---|
| offering a range of options (Morland et al., 2019) | |
| - Treatment components applied flexibly, depending on needs of client (Steinmatz and Gray, 2017) | |
| - Prioritize survivor voices, rights, perspectives in design of digital intervention (Emuzue, 2021) | |
| - Treatment-interfering cognitions [related to distance modality] may need to be addressed early in treatment (Valentine et al., 2020) | |
| - High satisfaction with services in part due to valuing being treated by therapist speaking native language and cognizant of values in native culture (Zheng and Grey, 2014) | |
| - Successful [distance therapy] requires two conditions: reliability of device, clients' willingness to access clinical service by distance (Zheng and Grey, 2014) | |
| - Therapeutic alliance formed quicker with preferred language and preferred modality, culturally sensitive validation (Zheng and Grey, 2014) | |
| - Critical to consider and problem solve from the beginning of treatment how to retain client(s), and to remain flexible as a provider (Banducci, 2021) | |
| ***Considerations for virtual sites (ie. satellite sites, counselling from home)*** | |
| - Satellite sites - warmth of supportive staff important, help with paperwork and routine things important, coordination and resource deployment between sites important (Azevedo et al., 2016) | ⇒ **The setting up of virtual sites, and considerations for survivor experiences of satellite side important** |
| - Allowing (satellite) crisis center staff to assist with facilitating initial contact between clients and therapists often increases clients receptivity to and comfort with psychotherapy (Steinmatz and Gray, 2017) | |
| - Helpful to have IT support at the proximal end (satellite sites) (Steinmatz and Gray, 2017) | |
| - Remind client to treat phone session as they would an in-person session (i.e. quiet room without distractions) (Sitz et al., 2021) | |
| - Create safe and calm virtual space for survivors to meet with advocates (i.e. calming background, appropriate lighting, space free of background noise for survivors to feel comfortable and safe discussing trauma), and encourage survivors to create comfortable space themselves (Voth Schrag et al., 2022) | |
| - Ensure survivors are given clear instructions and ongoing support on use of technology, provide information about selected virtual platforms, suggestions for how to create space to conduct video conferencing to foster clear communication (i.e. provide on agency website, or in initial intake session to allow for Q&A and support survivors in implementing) (Voth Schrag et al., 2022) | |
| - Challenges of conducting emotionally challenging and necessarily private work from home; need for separation between staff member's personal and family space and stressful work environment – should inform teleworking arrangements moving forward to prevent STS and burnout (Voth Schrag et al., 2020) | |
| ***Structuring distance sessions*** | |
| - First sessions- information gathering and rapport building (Steinmatz and Gray, 2017) | ⇒ **Distance sessions do not need to be replicative of traditional face-to-face sessions** |
| - Three initial sessions to collect background information and complete psychodiagnostic assessment (Banducci, 2021) | |
| - Consider structuring sessions differently (massing in beginning of treatment) (Valentine et al., 2020) | |
| - Shared decision-making tools during intakes or clinic orientations can help guide treatment planning process for treatment type and delivery modality (Morland et al., 2019) | |
| - New option of including peer support during in-vivo exposure exercises, in pre-treatment education, and during treatment (Valentine et al., 2020) | |

| | |
|---|---|
| - Sessions re-scheduled within same week whenever possible; minimize attrition by collecting additional contact information, making reminder calls for sessions, ensuring flexibility of scheduling (Gilmore et al., 2016) | |
| - Allow space and time for client to respond to your questions (Sitz et al., 2021) | |
| ***Cautions for distance counseling*** | |
| - Participation less likely if experiencing IPV from current partner (Stevens et al., 2015) | |
| - Survivors may worry about rights and choices when using impersonal data technologies to discuss sensitive and dangerous issues (Emuzue, 2021) | |
| - May need additional education about role of avoidance in symptom maintenance, how [distance modality] can reinforce avoidance (Valentine et al., 2020) | |
| - Clinicians should assess motivation to return after each session and discuss retention throughout course of treatment (Valentine et al., 2020) | |
| - Beneficial to inquire and discuss client concerns regarding [distance modality] (Steinmatz and Gray, 2017) | |
| - Further assessment of the varying reasons for CVT non-completion: avoidance, pragmatic barriers, negative treatment beliefs, reduced observation of clinical effectiveness, facility with technology, or modifications to therapist patient rapport would be illustrative (Valentine et al., 2020) | ⇒ **Need for assessment and strong communication with each survivor to determine (continued) individual candidacy for distance modality** |
| - Some work cannot be delivered online or to sufficient quality, harder to conduct risk assessments, harder to ensure no one else sees communication with client, make contact with clients, build rapport and follow up digitally (Baffsky et al., 2022) | |
| - Without face-to-face contact, more difficult for practitioners to assess risk (Cortis et al., 2021) | |
| - Miss non-verbal cues with remote, telehealth approaches (Cortis et al., 2021) | |
| - Empathic connection more difficult, assessing environments more challenging, technological challenges, boundary maintenance, fear related to who might be listening to a session or tracking participants movements electronically (VothSchrag et al., 2022) | |
| - Difficult to ascertain whether perpetrators are present during service delivery, difficulty recognizing signs of abuse and assessing risk, difficult to assess clients' circumstances and needs when contact is limited to phone calls, less confident to identify violence and its impacts (Cortis et al., 2021) | |
| ***Exclusion criteria*** | |
| - Presence of comorbid conditions with the potential to affect their ability to participate safely in and benefit from treatment: 1) active psychosis or dementia at screening, 2) suicidal ideation with clear intent, 3) current substance dependence (Gilmore et al., 2016) | |
| - Did not have an active psychotic or bipolar disorder and were not deemed at high risk for harm to themselves or others (Valentine et al., 2020) | ⇒ **Exclusion criteria: active psychosis or bipolar disorder, dementia, at risk of self-harm, suicidal, homicidal, substance dependence** |
| - Current guidelines caution against the use of telepsychiatry with suicidal patients, unless no other option is available (Thomas et al., 2005) | |
| - Acutely suicidal or homicidal individuals not eligible for telehealth services, pre-eminence of safety concern and unclear ability to manage such crises distally (emergency services appropriate until emergent need adequately addressed) (Steinmatz and Gray, 2017) | |
| - Unclear ability to manage distally someone so severely distressed they are at risk of self-harm, acutely suicidal or homicidal individuals not eligible for telehealth services, rather emergency resources in community more appropriate in | |

| | |
|---|---|
| meantime (Hassija and Gray, 2011) | |
| - Consider which patients will persist and how the clinician and system can encourage treatment completion (Banducci, 2021) | |
| - Have clear and informed discussion with patients regarding what trauma-focused treatment by distance involves and how treatment will fit into their lives, especially considering pandemic and stress-levels and day-to-day lives (Banducci, 2021) | |
| ***Technology discussions*** | |
| - HBT care delivered using Movi/Jabber software packages with standard internet connection to teleconference using federal government approved encryptions and are HIPPA compliant (Gilmore et al., 2016) | |
| - Veterans without a home computer received a tablet device with LTE connectivity and televideo encryption software (Gilmore et al., 2016) | |
| - Low bandwidth telehealth applications may improve connectivity in areas where internet speed is limited (Azevedo et al., 2016) | |
| - Telemental health technicians play pivotal role in making sure sessions go smoothly, scheduling sessions, ensuring clients feel comfortable with equipment (Azevedo et al., 2016) | |
| - Ever-evolving technology - important for TMH programs to expect changes and to be able to rapidly respond to them (secure exchange of confidential information), requires personnel and equipment resources (newer model computer, webcam, strong internet connection) (Azevedo et al., 2016) | ⇒ **Technology underpins survivor safety (which is paramount)**<br>⇒ **Importance of: secure, encrypted, HIPPA compliant software and hardware**<br>⇒ **Technology is continuously evolving, distance programs must rapidly respond with resources, IT support, investment**<br>⇒ **Have backup contact plans in place between survivor and counsellor to minimize disruption** |
| - Technology requirements made in-home TMH more difficult for clients with fewer means. Program made tablets available to clients without computer access (Azevedo et al., 2016) | |
| - Teleconference link used a T1 link between desktop computers with Polycom videoconferencing equipment and software; telephone and facsimile contact were also available during tele-conference sessions as back-up communication links (Thomas et al., 2005) | |
| - Secure, encrypted, videoconferencing-based technology (Polycom VSX3000 video- conferencing units) (Steinmatz and Gray, 2017) | |
| - Distal sites have high-speed Internet connection and the availability of a small, private room that could be used by clients for sessions<br>(Steinmatz and Gray, 2017) | |
| - Secure, encrypted videoconferencing technology (Hassija and Gray, 2011) | |
| - Technical difficulties are realistic possibility when providing services electronically - back-up plan for service provision should be discussed with the client to avoid interruption with service delivery (i.e., convert to phone if video interrupted) (Hassija and Gray, 2011) | |
| - clients and therapists should know how to contact one another via telephone during session in event of tech failure (Hassija and Gray, 2011) | |
| - Backup plan should be discussed with client to avoid interruption with service delivery (Steinmatz and Gray, 2017) | |
| - Store clients' correspondence in de-identified way (Baffsky et al., 2022) | |
| - Use HIPAA compatible platform for sharing of paperwork and worksheets (Sitz et al., 2021) | |
| - Utilize protected phone number (i.e. *67, Google voice, Doximity Dialer) to ensure confidentiality of phone numbers, potentially linking common phone number to virtual platform account to allow multiple therapists to long into account | |

| | |
|---|---|
| and take part in telehealth sessions with client (Sitz et al., 2021) | |
| - Technology safety is now part of job, where technology and internet access is critical for survivors and advocates; Need for encrypted laptops and tablets as well as a phone to call survivors from protected number (Voth Schrag et al., 2022) | |
| - Video conference platforms (Zoom, Web-ex, Go to Meeting); Phone calls; Video calling (Skype, Facetime) (Wood et al., 2020) | |
| - "Communication trail" through which perpetrator can see victim-survivor is seeking help (i.e. some video chat software stores automatically identifiable logs of calls between clients and practitioners) (Baffsky et al., 2022) | |
| - Use encrypted web-based video calls to avoid downloading phone apps that perpetrators might find (Cortis et al., 2021) | |
| **Pandemic-Specific Distance Conditions** | |
| ***Survivor Safety*** | |
| - Survivors may face inherent structural and practical barriers to accessing digitalized services while sheltered-in-place (internet connectivity issues, high-data burden, accessibility issues), impairs help-seeking, and pronounce disparities for rural, low-income, and older survivors (Emuzue, 2021) | ⇒ **New safety considerations, given sheltering-in-place**<br>• Scheduling – ask about safety and privacy<br>• Expand IPV screening and support to everyone<br>• Tech options for user security measures (stalker detection software, authentication and verification protocols)<br>• Counsellor/Survivor passcodes<br>• Introduce new ways to share information and seek privacy (i.e. chat function)<br>⇒ **Transformed and intensified violence due to pandemic conditions and stressors**<br>⇒ **New barriers to getting help**<br>⇒ **Technology means access to supports, but also introduces new and intensified threats to safety and security**<br>⇒ **Agency may be only private/safe place, consider** |
| - Changes to access to services due to COVID-19, remote type therapies and services likely to place many victims at risk for further violence by abuser, or may lack access to phones and internet due to financial hardships or coercive control (Kaukinen, 2020) | |
| - Emerging forms of technology-based abuse have spiked (online stalking, zoombombing, cyberbullying, doxing, sexualized trolling, nonconsensual pornography, coercive behaviors; stay-at-home directs facilitates interception and round-the-clock surveillance of social media and mobile devices (Emuzue, 2021) | |
| - COVID related stressors may simultaneously increase the risk of IPV while making potential to escape less feasible (Jarnecke and Flanagan, 2020) | |
| - Technology increases vulnerability to cyber-based violence (where abusive partners control, stalk or discredit their partners through texts, social media, or mobile applications) (Emuzue, 2021) | |
| - Increased monitoring by partner, professionals might apply increased flexibility with how and when they communicate with their client<br>(Jarnecke and Flanagan, 2020) | |
| - Providers should prioritize confidentiality during all parts of a visit, from scheduling to documenting (Ragavan et al., 2020) | |
| - Schedulers should ask adolescents if they have a comfortable space to speak and a safe phone where the virtual visit can be completed without being monitored (Ragavan et al., 2020) | |
| - Prioritize survivor privacy and safety, offer personalized real-time access to DV screening, risk awareness and support services (Emuzue, 2021) | |
| - Heightened need for discrete ways to access IPV services (personalized PIN numbers, disguised apps, code-word) (Jarnecke and Flanagan, 2020) | |
| - Expand IPV screening procedures across disciplines, create protocols for integrating screenings into telehealth visits to identify at-risk individuals (Jarnecke and Flanagan, 2020) | |
| - App designers and vendors should set up interface-level security measures that can distinguish abuser from victim | |

| | |
|---|---|
| based on cues; covert authentication and verification protocols (emergency exit buttons, app lockdown, data dump after failed password entry), also, passcodes; Special stalker detection software (Emuzue, 2021) | **keeping open with strict protocols as last option**<br>⇒ **Individualized safety planning, confirm client location at outset, use code words** |
| - When completing AYA virtual visits with parents or other parties present, clinicians should set up expectations regarding the importance of having time to speak alone with adolescent, aligned with best practices during in-person visits (Ragavan et al., 2020) | |
| - Although conducting visits virtually is safer for physical distancing, AYAs should be given a choice about completing a visit virtually or in-person (for some AYAs, healthcare centers may be one of the only available safe places) (Ragavan et al., 2020) | |
| - If an adolescent does not feel safe talking during a virtual visit, providers may consider using the chat function, as long as the adolescent's phone or computer is not being monitored and conversations are not being saved (Ragavan et al., 2020) | |
| - Practitioner concerns about client privacy and confidentiality working at home and in isolation (Cortis et al., 2021) | |
| - Limited opportunity with pandemic to arrange code words or in-depth safety plans (Cortis et al., 2021) | |
| - Children may be present during intake or screening (Cortis et al., 2021) | |
| - Collaborate with survivor to develop personalized plan to address immediate, specific risks, with aim to reduce extent and impact of violence and abuse moving forward (Wood et al., 2020) | |
| - Survivors, especially from Black and Brown communities, may not view police or other formal first responders as safe or supportive avenues to address potentially violent situations (Wood et al., 2020) | |
| - Safety planning is inherently individually focused, not a one-size-fits-all set of solutions, but unique mix of strategies needed for each situation (Wood et al., 2020) | |
| - Confirm client location at outset of each session should emergency protocols need to be enacted during session, and to confirm state licensure compliance (Sitz et al., 2021) | |
| - Use code words in text and phone communications to signal risks while perpetrators present (Cortis et al., 2021) | |
| ***Counsellor and Agency Considerations*** | |
| - Agency staff working under pandemic conditions face adverse psychological stress, compassion fatigue, burnout; May need intentional acts of self-care and separation from work to continue (Emuzue, 2021) | ⇒ **Staff taking on brunt of mismatch between demand and capacity for transitioning to distance, require working flexibility, self-care, peer support, resources (training, IT support, protective equipment)**<br>⇒ **Nature of work has changed and intensified, resulting in occupational stress, interpersonal tensions** |
| - Support mental well-being of frontline staff: (1) develop self-care plans; (2) group support sessions; and (3) flexible work schedules to accommodate caregiver responsibilities, including sick leave and paid time off (Ragavan et al., 2020) | |
| - Burden on agency staff to appraise and become familiar with safe use of new technologies (Emuzue, 2021) | |
| - Overextended bandwidth, device/subscription requirements, IT issues, data privacy, data mining worry (Emuzue, 2021) | |
| - More help-seeking during natural disasters, overburdened services, funding cuts (government funds diverted for other emergent needs) (Emuzue, 2021) | |
| - All frontline staff should have appropriate personal protective equipment and training in tele-health strategies to help them continue fostering connections with clients (Ragavan et al., 2020) | |
| - Be aware of personal, technological, other barriers to providing trauma-focused treatment during pandemic; ensure plan is in place to address these barriers prior to suggesting treatment (Banducci, 2021) | |
| - Rapid implementation, increased demand for services coupled with changing service delivery resulted in increased | |

| | |
|---|---|
| stress for practitioners who are feeling stretched and overwhelmed, working extended hours to meet increasingly complex needs of growing clientele (Baffsky et al, 2022) | |
| - Challenging for frontline workers to take time off putting them at risk of burnout (Baffsky et al., 2022) | |
| - Professional isolation and increased risk of vicarious trauma (Baffsky et al., 2022) | |
| - Fewer cancellations, more check-ins due to concerns for elevated risks posed by pandemic (Baffsky et al., 2022) | |
| - Capacity to pay for technology, infrastructure, and training needed as staff transitioned to work from home (Cortis et al., 2021) | |
| - New offerings of online support and expanded contact hours, increasing demands on practitioners, including extra tasks (checking on clients more regularly, choosing suitable technology and apps, ensuring clients had useable devices, assisting clients with online communications, discussing safe use of technology (Cortis et al., 2021) | |
| - Online/remote delivery involved considerable extra work for practitioners, added stress when working away from usual collegial supports takes toll on well-being (Cortis et al., 2021) | |
| - Virtual work is more task oriented (Cortis et al., 2021) | |
| - Technology-mediated service delivery constituted new way of working adopted out of necessity not choice, and without reference points of practice guidelines or evidence base (Cortis et al., 2021) | |
| - Additional support outside of regular sessions necessary to retain client (Banducci, 2021) | |
| - Shifts in practice by distance include: more frequent contact, increased focus on emergent or crisis needs-in-the-moment advocacy (Voth Schrag et al., 2022) | |
| - New technology needs and training associated with offering virtual services (Voth Schrag et al., 2022) | |
| - Frequent shifts in procedures, shifts in 'who is in the office', tensions between work-from-home and site-based staff (Voth Schrag et al., 2022) | |
| - IPV/SV workforce has had shift both practice model and approach in pandemic, creating additional risks for occupational stress in already strained workforce (Wood et al., 2020) | |
| - New sets of resources to know and develop to refer clients to because of the pandemic (Wood et al., 2020) | |
| - Ensure staff has access to resources necessary to carry out work (i.e. hazard pay, counseling, assistance with material and resource support such as childcare) essential to minimizing stress impacts in workforce (Wood et al., 2020) | |
| ***Changes to access*** | |
| - Need for change in the way in which awareness campaigns have provided information to IPV victims and bystanders (cannot rely on public spaces) - diverse social media outlets should seek to identify ways to reach a wider audience during city lockdowns and stay-at-home orders (Kaukinen, 2020) | ⇒ **Cannot rely on traditional channels of mass communication, referrals**<br>⇒ **Cannot assume survivor access to technology**<br>⇒ **Pandemic has changed patterns of access** |
| - Virtual relationship-building assumes adolescents have access to stable Internet, data access, phone and computer equipment to sustain relationships – points to advocacy for access to free or low-cost technology (Ragavan et al., 2020) | |
| - Treatment engagement, including elevated rates of dropout and low likelihood of trauma-focused treatments being initiated need to be considered and intervened upon (Banducci, 2021) | |
| - With virtual service model, agencies need to focus on "virtual front door", taking same care as in-person survivor comfort with additional attention to safety and digital security (Voth Schrag et al., 2022) | |
| - Digital divide, reduced availability and accessibility of support services for those most vulnerable due to inequities in digital participation (Baffsky et al., 2022) | |
| - Practitioners working with limited referral options, as other services closed or reduced capacity (Cortis et al., 2021) | |

| | |
|---|---|
| - Pandemic-driven changes in patterns of [service] demand, including increased requests for support and greater complexity amongst people using service, more access from people without history of violence (Cortis et al., 2021) | |
| - Ensuring access for people with language barriers, those without technology, parents supporting children at home with remote learning (Cortis et al., 2021) | |
| - Survivors may feel safer accessing services online, especially for those stigmatized for seeking services, more comfortable with anonymity of virtual services, for example hard to reach populations or populations that experience marginalization may especially benefit from virtual service expansion  (Voth Schrag et al., 2022) | |
| - Continuing virtual services and potentially adding them as part of an a la carte service model (Voth Schrag et al., 2022) | |
| - Safe and effective virtual services will enhance reach of IPV/SA services beyond pandemic (Wood et al., 2020) | |
